# Patient Re-Identification Based on Deep Metric Learning in Trunk Computed Tomography Images Acquired from Devices from Different Vendors

**DOI:** 10.1007/s10278-024-01017-w

**Published:** 2024-02-16

**Authors:** Yasuyuki Ueda, Daiki Ogawa, Takayuki Ishida

**Affiliations:** 1 Division of Health Sciences, Graduate School of Medicine, Osaka University, 1-7 Yamadaoka, Suita, Osaka 565-0871 Japan; 2 School of Allied Health Sciences, Faculty of Medicine, Osaka University, 1-7 Yamadaoka, Suita, Osaka 565-0871 Japan

**Keywords:** Deep metric learning, Medical imaging, Patient re-identification, Augmentation technique, Convolutional neural network

## Abstract

During radiologic interpretation, radiologists read patient identifiers from the metadata of medical images to recognize the patient being examined. However, it is challenging for radiologists to identify “incorrect” metadata and patient identification errors. We propose a method that uses a patient re-identification technique to link correct metadata to an image set of computed tomography images of a trunk with lost or wrongly assigned metadata. This method is based on a feature vector matching technique that uses a deep feature extractor to adapt to the cross-vendor domain contained in the scout computed tomography image dataset. To identify “incorrect” metadata, we calculated the highest similarity score between a follow-up image and a stored baseline image linked to the correct metadata. The re-identification performance tests whether the image with the highest similarity score belongs to the same patient, i.e., whether the metadata attached to the image are correct. The similarity scores between the follow-up and baseline images for the same “correct” patients were generally greater than those for “incorrect” patients. The proposed feature extractor was sufficiently robust to extract individual distinguishable features without additional training, even for unknown scout computed tomography images. Furthermore, the proposed augmentation technique further improved the re-identification performance of the subset for different vendors by incorporating changes in width magnification due to changes in patient table height during each examination. We believe that metadata checking using the proposed method would help detect the metadata with an “incorrect” patient identifier assigned due to unavoidable errors such as human error.

## Introduction

A standard medical image formatted Digital Imaging and Communication in Medicine consists of the medical image and the header. The metadata in the header contain relevant patient-specific information such as name, age, sex, and date of birth, along with technical data and parameters, such as the device used to generate images. Patient identification errors can be attributed to manual processes by healthcare providers, such as verbal verification of the patient, and images with incorrect metadata stored in picture archiving and communication systems (PACS) may remain unnoticed until they impact patient care. In radiologic interpretation, radiologists generally confirm details in the metadata and identify the patient in the image. Therefore, if the image metadata are from a different patient, the image will be acquired for a different patient, resulting in patient misidentification [1–6]. In the 2021–2022 annual report on the statutory notifications of significant accidental and unintended exposures for the “Ionising Radiation (Medical Exposure) Regulations 2017” by the Care Quality Commission in the United Kingdom, the most common type of error was a patient receiving an examination intended for another patient [7]. According to Morishita et al. [3], the average rate of misfiled radiography cases at a hospital is 0.075%. In another study [8], the estimated near-miss wrong-patient event rate for misfiling radiologic examination cases was 0.002%. The main risk associated with patient misidentification in clinical radiography is the unnecessary exposure of the wrong patient to radiation, which can result in serious incidents or accidents, such as performing surgery on the wrong patient [5].

Image retrieval is a computer vision task that aims to find images similar to an image query from a large-scale image database such as PACS [9–11]. Recently, as an applied technology of content-based image retrieval to solve patient misidentification, image analysis techniques that link the correct patient information with an image, called patient re-identification, have shown promising results for X-ray images [2, 4, 12, 13–20], two-dimensional scout computed tomography (CT) [21, 22], three-dimensional (3D) scout magnetic resonance images [23], and 3D CT [24] images. The main challenge in patient re-identification is the extraction of valid feature vectors (representations) from medical images in a manner that increases interpatient differences while decreasing intra-patient variations.

Even in the deep learning era, various deep convolutional neural network (DCNN) methods trained on medical imaging datasets experience domain shifts owing to clinical variations [25–28]. This raises a concern regarding the generalization capacity of deep learning models. Hence, decreasing the domain shift is important in effectively applying deep learning-based methods to patient re-identification.

Many current methods using medical imaging datasets are based on the assumption that the training and test distributions consist of identical or few domains [25–28]. On chest X-ray images, a patient re-identification method using deep metric learning considering two domains, the view position between the anteroposterior and posteroanterior, has shown promising results [12].

**Fig. 1:**
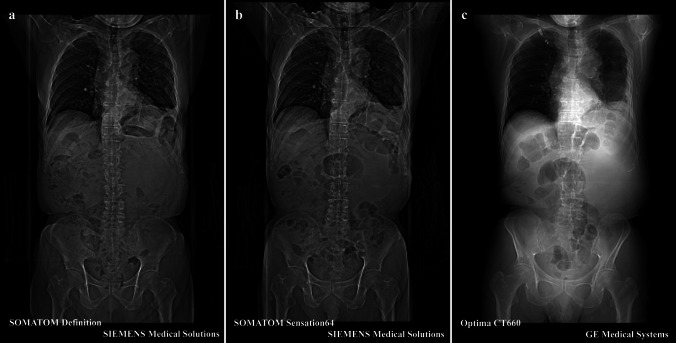
Examples of images acquired using devices from different vendors. Three scout images in a patient were acquired using **a** SOMATOM Definition, **b** SOMATOM Sensation64 (SIEMENS Medical Solutions), and **c** Optima CT660 (GE Medical Systems)

Additionally, in the annual report mentioned above, the highest proportion of notifications from diagnostic imaging was from CT in 2021–2022. In previous studies, the patient re-identification techniques using scout images revealed significant potential for verifying a patient’s identity [21–23]. In clinical scout CT, the scout scan is usually acquired before the main scan, and the trunk is typically scanned in the frontal view position. Every CT device acquires a scout CT image with slightly different geometries of the source-to-image-receptor distance (SID) and image processing. Most clinical CT devices have an SID of approximately 100 cm, implying the absence of any major changes in the magnification of each CT device at the object. In addition, each CT examination acquires a scout CT image with a slightly different geometry of the object (here, the patient) from that of the image-receptor distance depending on the table height. Although healthcare workers usually position the scanner table such that the patient is at the center of rotation, the table height is not necessarily the same as in the previous CT scan [29–31]. Therefore, the magnification at the transverse end of the scout CT image in each CT scan varies slightly [29, 30]. Moreover, differences in processed images may be noticeable in each scout CT image between devices from different vendors as shown in Fig. 1 because of how each vendor applies distinctive image processing techniques, such as image contrast correction and dynamic range compression [25, 26]. Therefore, scout CT images may have domain shifts due to different image acquisition conditions and image processing depending on the scanner vendor and patient positioning reproducibility [25–28].

This study aimed to (a) develop a feature extractor trained on clinical trunk scout CT images using deep metric learning and (b) quantitatively evaluate the outcomes in patient re-identification accuracy using the proposed method on trunk scout CT image datasets acquired using scanners from multiple vendors.

## Materials and Methods

### Outline of the Proposed Method

**Fig. 2:**
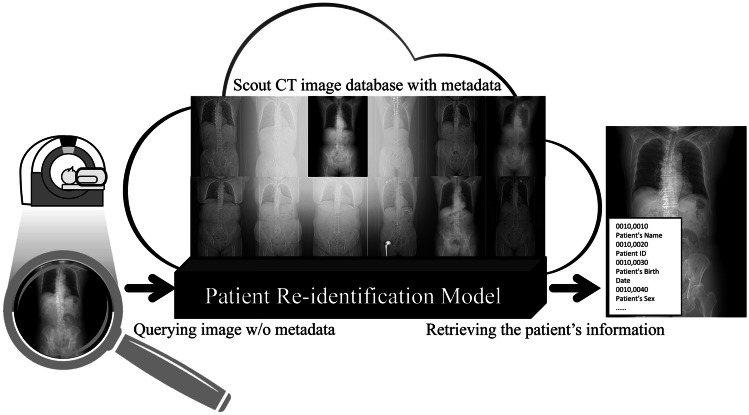
Proposed system for patient re-identification

The proposed patient re-identification scheme using a deep feature extractor is illustrated in Fig. 2. The workflow comprises three steps: (i) image acquisition and preprocessing, (ii) feature extraction, and (iii) similarity score matching. The first step involves acquiring a scout CT image from the patient’s examination using a routine trunk CT scan. Next, the feature extractor trained by the proposed DCNN model extracts feature vectors using a universal patient representation from the scout CT image without metadata. In the final step, the most similar scout CT image from the stored dataset is determined as a similarity index based on the cosine similarity of the feature vector extracted between the scout CT image and each stored clinical dataset. The patient’s information on the most similar image is assigned to all images from that CT scan, including the main scan.

### Datasets

This retrospective, observational study was approved by the Institutional Review Board of our institution (approval number 21064–4). Written informed consent was not required owing to the study’s retrospective design. All procedures conducted in this study conformed to the tenets of the Declaration of Helsinki.

This study used scan data from 5540 patients who underwent chest–abdomen–pelvis CT between March 2015 and May 2016 at Yamaguchi University Hospital in Yamaguchi, Japan. Patients aged < 20 years were excluded. All scout scans were performed using the following three CT devices: SOMATOM Sensation64, SOMATOM Definition (SIEMENS Medical Solutions, Forchheim, Germany), and Optima CT660 (GE Medical Systems, Waukesha, WI, USA). Several radiologists have interpreted the series of CT images to which the scout image used in this study belongs. Here, interpretation includes comparing the current examination with the patient’s previous examination and other examinations using different modalities. Furthermore, all patient IDs before anonymization have been confirmed to match those on file in the hospital information systems. Table 1 presents the patient demographics in the dataset used for the training, validation, and testing of the DCNN used by the proposed method. Table 2 shows the scan conditions for trunk scout CT imaging in the dataset.

**Table 1:** Patient characteristics

	All	Training	Validation	Test
Number of images	8828	1997	732	6099
Number of patients	5540	732		4808
Age (years)				
Mean ± SD	67 ± 13			
Range	20–100			
Sex				
Male	3077			
Female	2463			

*SD* standard deviation

**Table 2:** Scan characteristics

	SOMATOM Definition	SOMATOM Sensation64	Optima CT660
Vendor	SIEMENS	SIEMENS	GE
Number of images	3057	3997	1774
Tube voltage (kV)	120	120	120
Tube current (mA)	20–36	20–35	20
Pixel spacing (mm^2^) (columns × rows)	2.0 × 2.0	2.0 × 2.0	0.55 × 0.60
Table height (mm)			
Mean ± SD (mm)	141 ± 14	147 ± 14	135 ± 13
Range (mm)	100–198	95–223	88–188
Physical width of the image measured at the rotation center			
Detector size (mm)	0.6	0.6	0.625
Field of view (mm)	560	560	530

*SD* standard deviation

The dataset used for training and validation included 2729 images from 732 patients acquired from at least three trunk scout CT scans using one of the three CT devices; they were used either for training or validation, but not both. Out of the 2729 images, 1997 were used for training and 732 for validation of the trained model. The test dataset included 6099 images of 4808 patients acquired from less than three trunk scout CT scans in either of the three CT devices and were not included in the training or validation datasets. The images used in the test were those from the earliest examination date or a single examination (4808 images) of 4808 patients as the baseline images and the second examination date (1291 images) as the follow-up images. Pixel spacing of all images was resampled to 2.0 × 2.0 mm^2^ using bicubic interpolation and cropping the central 256 × 384 (columns and rows) pixels, and the bit depth was rescaled linearly down to 8 bits.

### DCNN Learning

EfficientNet [32] is a model of convolutional neural networks that was proposed in 2019. EfficientNet adopts mobile inverted bottleneck convolution (MBConv) [32–34], which is similar to MobileNetV2 [33] and MnasNet [34]. EfficientNet incorporates a compound scaling system that uniformly scales each dimension with a fixed set of scaling coefficients. EfficientNetV2 [35] was launched in 2021, featuring a smaller model and faster training method than its predecessor, EfficientNet. To address the issue of large image size and its impact on memory usage, EfficientNetV2 uses FixRes [36] to reduce image size for training without any post-training layer fine-tuning. EfficientNetV2 replaces MBConv with Fused-MBConv [37] to better use mobile or server accelerators and uses a single 3 × 3 convolution instead of depth-wise 3 × 3 convolution and expansion 1 × 1 convolution. To find the optimal combination of MBConv and Fused-MBConv, a training-aware Neural Architecture Search [34, 38] is proposed. EfficientNetV2 also uses modified progressive learning and training with different image sizes that adjust regularization intensity as needed and solve the issue of reduced accuracy resulting from changing image sizes during training [39].

In this study, the feature extractor was trained using the original EfficientNetV2-L [35] backbone, which outputs 1280-d of feature vectors. The classifier used for training consists of a nonlinear fully connected (FC) layer and a metric learning layer. The nonlinear FC layers comprise four sequential layers: a linear layer, rectified linear unit activation function, batch normalization layer, and linear layer. Additionally, we introduced AdaCos [40], a deep metric learning technique [41], to control the dimensionality reduction complexity while preserving each patient’s characteristics. AdaCos can automatically determine hyperparameters and perform deep metric learning without additional tuning steps. Feature vectors were extracted from the trained feature extractor to re-identify the examined patients from the scout CT images of the trunk.

Deep learning was performed using a computer with a Tesla P40 (NVIDIA, Santa Clara, CA, USA) graphics processing unit, EPYC 7302P 3.0 GHz (Advanced Micro Devices, Inc., Santa Clara, CA, USA) central processing unit, and 32 GB of random-access memory. Python 3.11.4 and PyTorch 2.0.1 + cu117 were used to perform the DCNN training, validation, and testing using the element-wise adaptive sharpness-aware minimization optimizer [42] (base optimizer SGD with a momentum of 0.8 and a weight decay of 0.002) with a neighborhood size (rho) of 2.0, batch size of 20, loss function of smooth cross entropy with a label smoothing of 0.05, and a learning rate scheduler of cosine decay (initial-last, 0.005–0.0005) without warmup.

### Data Augmentation

In this study, data augmentation for training images consisted of four sequential layers: random perspective, random rotation, random scaling for the transversal direction, and cropping on the center (see Appendix).

The height of the patient’s table is not always the same for baseline and follow-up CT scans. As the table height changes, the magnification changes at the transverse of the scout CT images owing to geometric effects [29–31]. In this study, to make the neural network more robust to image changes with patient table height variability, we proposed an augmentation technique, RandomTransversalScaling, which provides the effect of randomly varying the height of the patient table. The image width was randomly rescaled, and the magnification toward the image height remained fixed during the proposed data augmentation.

### Re-Identification Performances of the Proposed Method

The cosine similarity between the local 1280-d features in the baseline and follow-up scout CT images was used as the similarity index. The cosine similarity ranged from − 1 to 1 and was used to determine whether the patient pair corresponded to the same patient. The re-identification performance of the proposed method was evaluated using the test dataset (baseline, 4808 images of 4808 patients and follow-up, 1291 images of 1291 patients who underwent baseline examination). This study evaluated the top-1 and top-10 accuracies (ACC1 and ACC10) in the entire test dataset and two of its subsets, as shown in Table 3, under the combinations of the two vendors of each CT device used at baseline and in each follow-up examination. The top-K accuracy computes the proportions where the number of correct patient’s image is among the number of the top-K patient’s image, ranked by similarity index. The ACC1 is the ratio obtained between the pairs with the highest cosine similarity, whereas the ACC10 is the ratio obtained between the pairs within the top-10 higher cosine similarities. Both ACC1 and ACC10 ratios were calculated in all comparisons among the 4808 patients and related pairs. The calculation time of the test process using the proposed method was approximately 10 min for comparing 4808 × 1291 patients. However, the GPU memory required approximately 7 min to read the images of 4808 patients, and the calculation cost for clinical application could be shortened by calculating the feature vectors in advance.

**Table 3:** Two subsets (same vendor and different vendors) in the test dataset

Name of subset	CT device		Number of patients	
	**Baseline**	**Follow-up**		
Same vendor	S1	S1	169	887
	S1	S2	166	
	S2	S1	220	
	S2	S2	263	
	G1	G1	69	
Different vendors	S1	G1	57	404
	S2	G1	93	
	G1	S1	114	
	G1	S2	140	
Total				1291

*CT* computed tomography, *S1* SOMATOM Definition (SIEMENS Medical Solutions), *S2* SOMATOM Sensation64 (SIEMENS Medical Solutions), *G1* Optima CT660 (GE Medical Systems)

### Statistical Analysis

The ACC1s calculated for two subsets (same vendor and different vendors) from the test dataset were statistically compared using a *Z*-test to compare two unpaired proportions. Subsequently, the ACC1s calculated with and without the proposed data augmentation technique on different vendor subsets were statistically compared using McNemar’s chi-squared test to compare the two paired proportions [43].

## Results

**Fig. 3:**
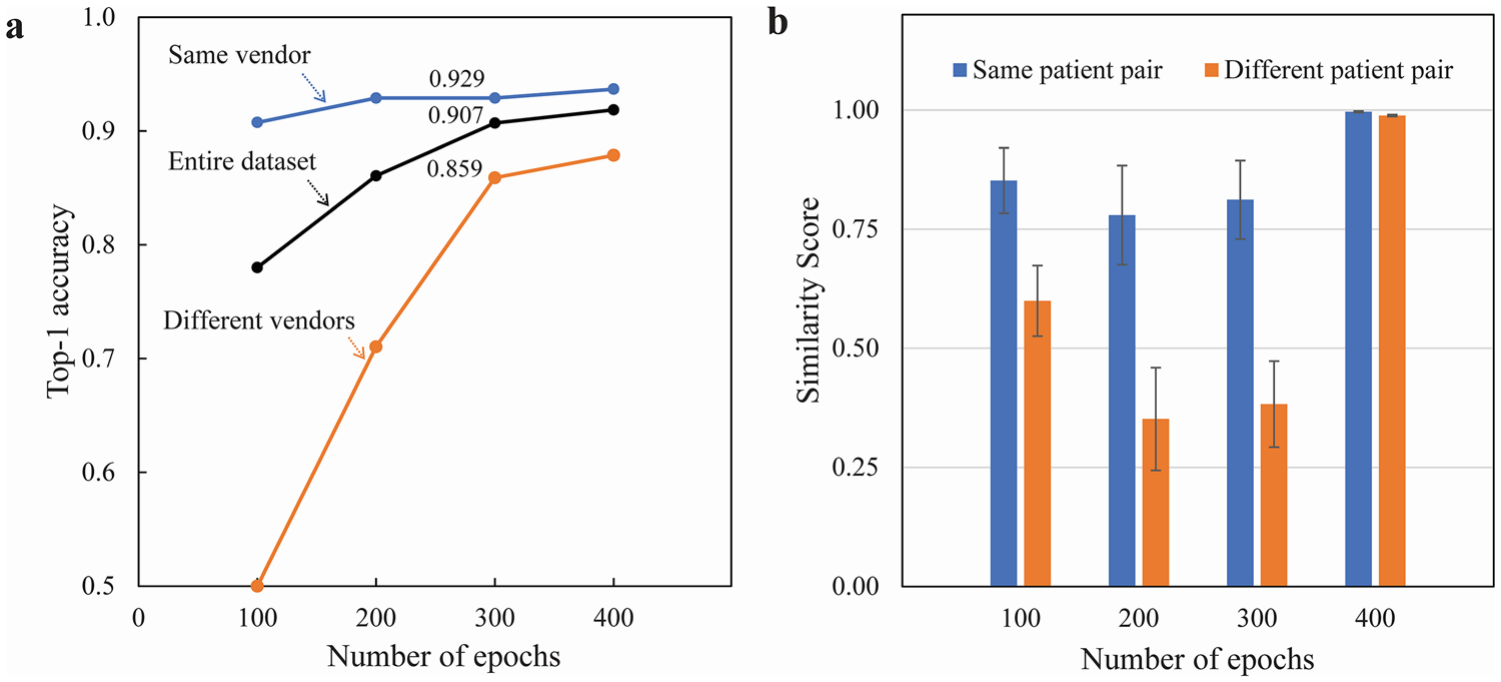
Top-1 accuracy performance and similarity score transition for the number of epochs in the proposed method. **a** Relationship between the number of epochs and top-1 accuracy for the entire dataset, same vendor, and different vendors. The black, blue, and orange solid lines represent the entire dataset, the same vendor, and different vendors, respectively. **b** Relationship between the number of epochs and the average similarity score for the same patient and different patients in the entire dataset. The blue and orange bars represent the same patient and different patients, respectively. Error bars represent standard deviations. Same vendor, a subset that consists of image pairs from scanners from the same vendor; Different vendors, a subset that consists of image pairs from scanners from different vendors; Same patient pair, similarity scores calculated from the same patient; Different patient pair, similarity scores calculated from different patients

Figure 3 shows the ACC1 performance and similarity score transitions for the number of epochs in the proposed method. ACC1 increased significantly as the number of epochs increased for the entire dataset and different vendors (*p* < 0.01). For the same vendor, ACC1 increased significantly when comparing 100 and 200 epochs; however, ACC1 showed no increase from 200 to 300 epochs. Figure 3a shows the transition of ACC1 for the number of epochs using the proposed method on the test dataset: the entire dataset and two subsets. The ACC1 performance on a subset comprising image pairs from the same vendor (blue line) was 0.9 or higher for all ranges of epochs and achieved a value of 0.929 at 300 epochs. The ACC1 performance in a subset comprising image pairs from different vendors (orange line) also improved as the number of epochs increased, achieving a value of 0.859 at 300 epochs. Figure 3b shows the transition of the similarity score for the number of epochs using the proposed method in the test dataset. At 300 epochs, the distributions of the similarity scores calculated for the same patient (blue bars) and those for different patients (orange bars) did not overlap. However, at 400 epochs, distributions for the same and different patients were concentrated around 1.0 with similarity scores.

**Fig. 4:**
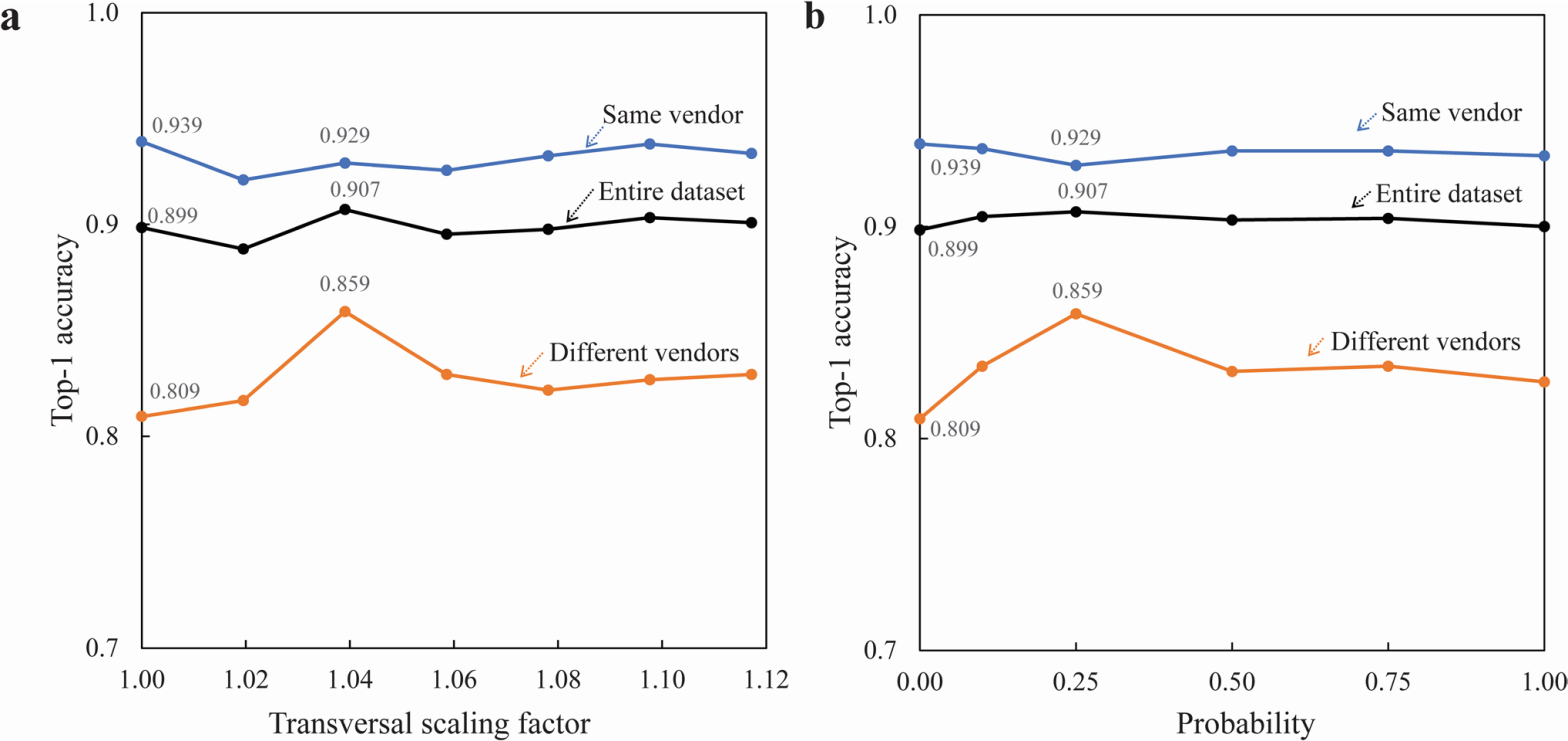
Top-1 accuracy performance using the proposed data augmentation method in the entire dataset and two subsets. **a** The relationship between the transversal scaling factor and top-1 accuracy. **b** The relationship between the probability of the image being transformed and top-1 accuracy

Figure 4 shows the ACC1 transition in the proposed method for RandomTransversalScaling for the entire dataset and two subsets by varying (a) the transversal scaling factor and (b) the probability of the image being transformed. Applying the proposed data augmentation method slightly improved the performance of ACC1 from 0.899 (without applying this augmentation) to 0.907 (applying this augmentation with the best performance) in the entire dataset; however, the improvements were not significant (*p* = 0.138). Furthermore, the performance of ACC1 improved from 0.809 (without applying this augmentation) to 0.859 (applying this augmentation with the best performance) in a subset of image pairs from different vendors (*p* < 0.01). However, for the transversal scaling factor of 1.04, where maximum performance was observed, a significant difference (*p* < 0.01) was observed between the two subsets from the same vendor (ACC1 0.929) and different vendors (ACC1 0.859).

**Fig. 5:**
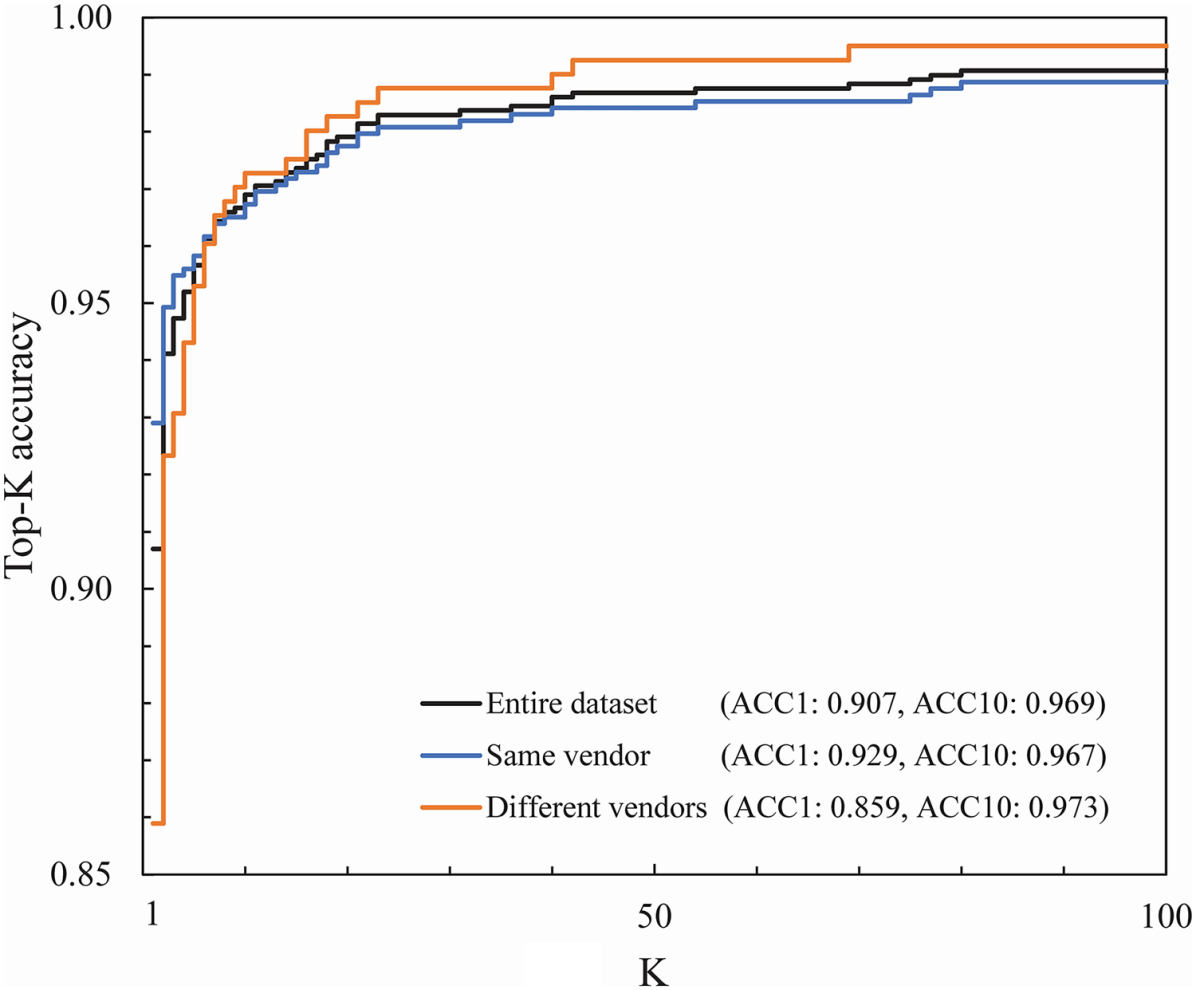
Comparison of CMC curve performances for the test dataset (entire, same vendor, and different vendors). Top-K accuracy shows the proportions where the correct patient’s image is among the top-K patient’s images ranked by the similarity index. CMC, cumulative match characteristic; Same vendor, a subset that consists of image pairs from scanners from the same vendor; Different vendors, a subset that consists of image pairs from scanners from different vendors

Figure 5 shows the cumulative match characteristic (CMC) curves after 300 epochs, with a transversal scaling factor of 1.04 and a probability of 0.25 for image transformation. The ACC10s for the entire dataset, same vendor, and different vendors increased from each corresponding ACC1 value to 0.969, 0.967, and 0.973, respectively.

**Fig. 6:**
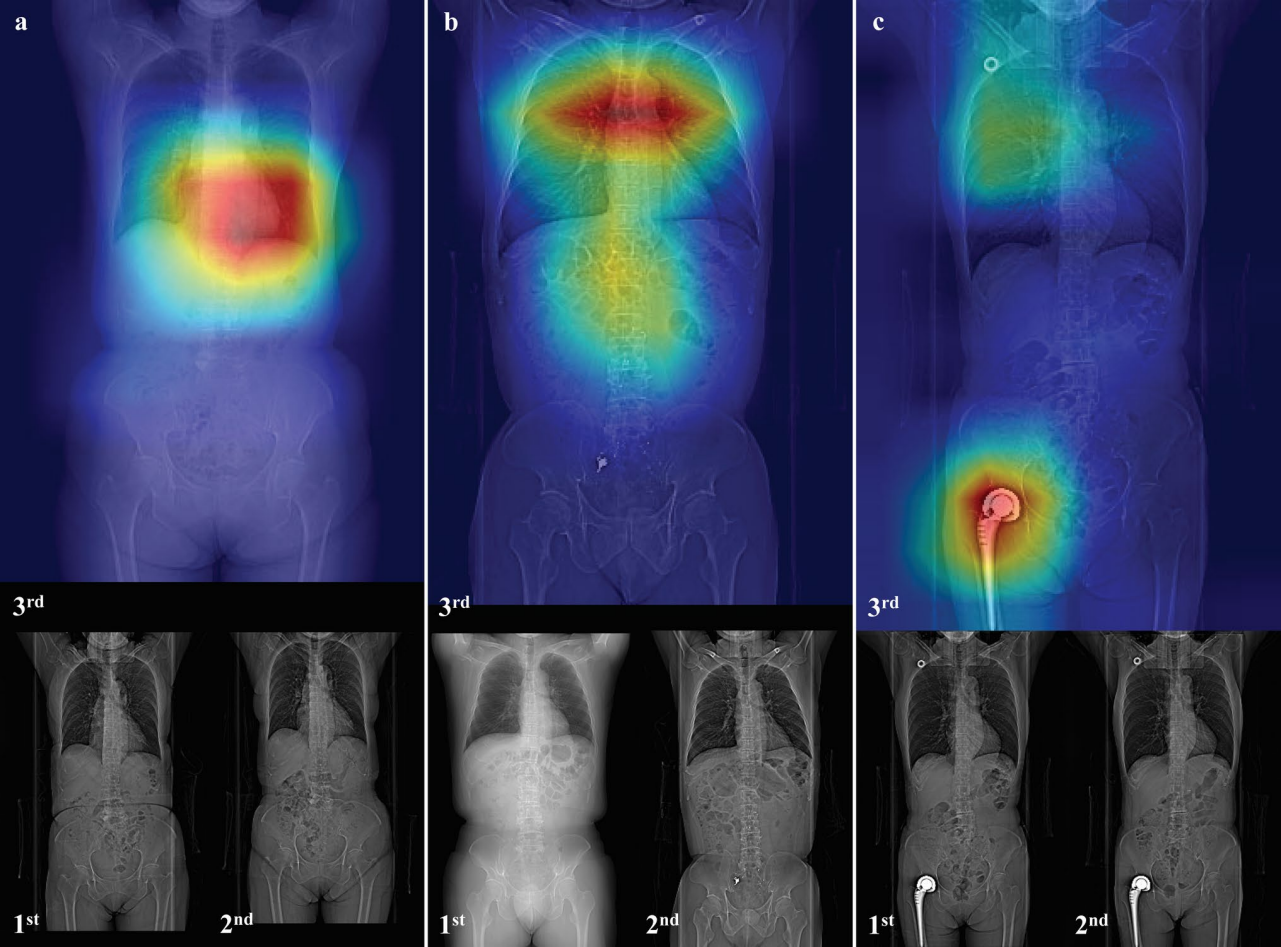
Visualization of an important region that identifies the patient using the trained model of the proposed method using Grad-CAM. Images of three patients (**a**–**c**) with three images each in the order of scan time, two images (first and second) from the training dataset, and one image (third) from the validation dataset overlaid on the Grad-CAM heatmap. **a** Image of a patient whose training (SIEMENS Medical Solutions) and verification images (GE Medical Systems) are obtained from scanners from different vendors. **b** Image of a patient with an implantation device (the second and third images show stent placement and coil in the abdominal-pelvic region). **c** Image of a patient with an implanted device (total hip arthroplasty implant and central venous port)

Figure 6 shows an important area visualization that identifies the patient using a model trained based on the proposed method using Grad-CAM [44]. Grad-CAM generates heat maps of local features with patient specificity and superimposes them on the original scout CT image to allow visualization of important regions for patient re-identification.

**Fig. 7:**
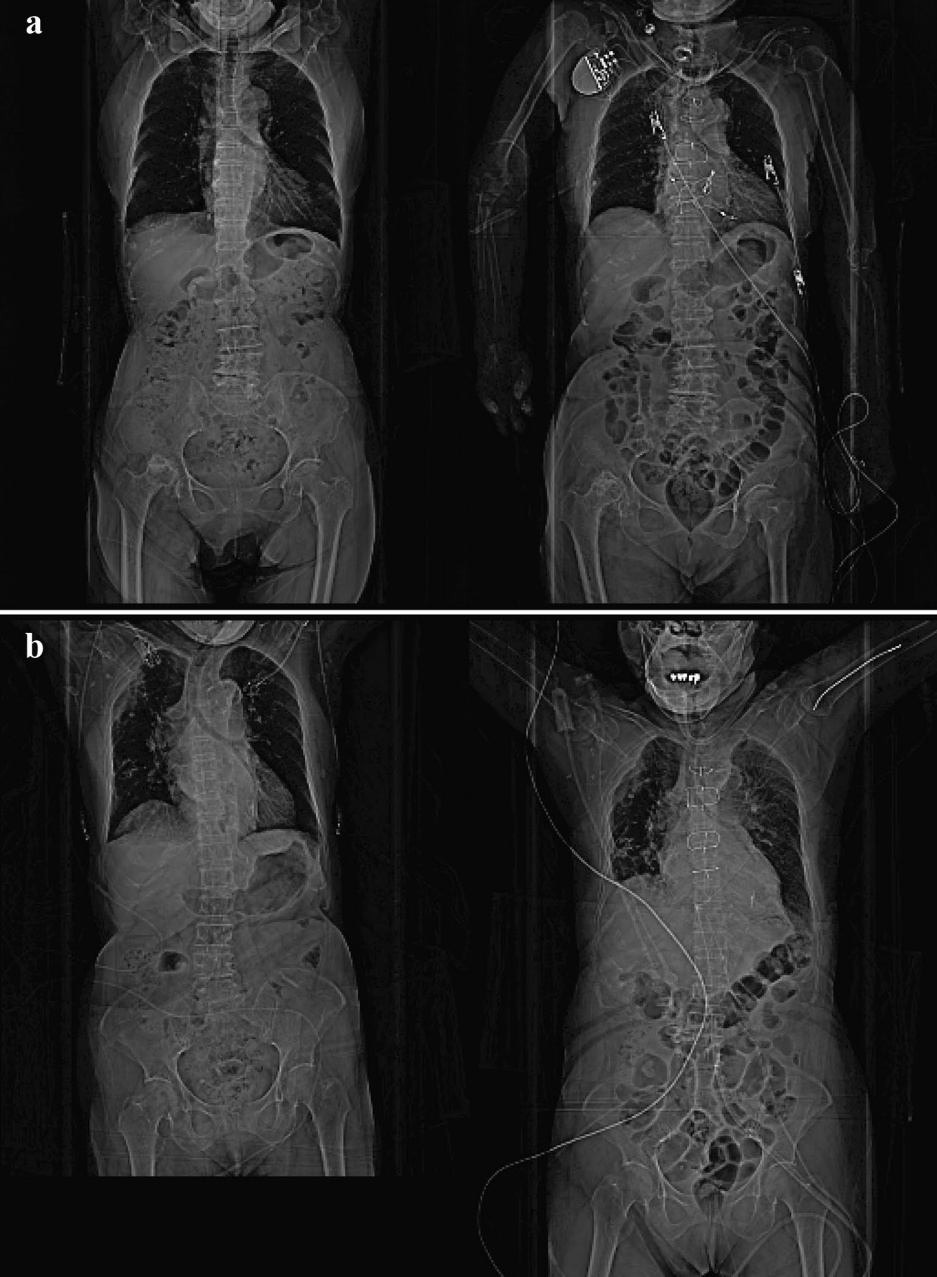
Examples of top-10 error patient-pairs. Example demonstrating top-10 error evaluation in the test dataset due to **a** arm position, both arms raised or not; intestinal gas, have fullness or not; **b** scan range, neck region included or not, intestinal gas, have fullness or not; and **c** lung condition, deep inspiration or not. Follow-up (right) and baseline (left) scans of single patients. The achieved similarity indexes were (a) 0.594 and (b) 0.685

The top-K error is the ratio of times the classifier failed to include the proper class among its top-K guesses; for the proposed method, this occurred 40 times out of 1291 guesses. Table 4 shows the top five reasons for each image pair resulting in a top-10 error. The most common reason was that the scan range differed between the two scout CT images. Next, lung condition, intestinal gas, scanner vendor, and arm position exhibited decreasingly high frequencies in that order. Figure 7 shows examples of same patient image pairs that resulted in a top-10 error.

**Table 4:** Top five reasons for each image pair resulting in a top-10 error

Reasons	Proportion
Scan range	0.500
Lung condition	0.400
Intestinal gas	0.325
Scanner vendor	0.275
Arm position	0.225

## Discussion

This study proposed an image-matching method using a DCNN feature extractor to re-identify examined patients using clinical scout CT images. A novel DCNN feature extractor, trained using a real clinical dataset of scout CT images, performed a dimensional reduction to 1280-d while retaining each patient’s characteristics. The re-identification performance using the proposed data augmentation scheme, adding a transformation of random scaling in the transversal direction, was significantly better for the subset of devices from different vendors in 300 epochs (ACC1 + 0.0495, *p* < 0.01).

Although a slight degradation was observed during the evaluation of subsets from the same vendor (ACC1 − 0.0101), the proposed model already achieved high performance (ACC1 of 0.9 or more) with or without RandomTransversalScaling. The ACC1 performance for the entire dataset was increased slightly (ACC1 + 0.00852); however, there were no significant differences.

Scout CT images have differences in geometry due to variations in magnification along the transverse direction, depending on the table height set during patient positioning. Furthermore, such geometric differences are more noticeable in devices from different vendors. We believe the ACC1 performance using our proposed data augmentation method with RandomTransversalScaling was improved.

The optimized hyperparameters that yielded the highest ACC1 value were 300 epochs, a transversal scaling factor of 1.04, and a probability of 0.25 that an image was transformed. When the number of epochs was optimized, the ACC1 performance improved, even when the number of epochs increased from 300 to 400. However, the distributions of performance for the same patient and different patients were concentrated around a similarity score of 1.0 as shown in Fig. 3b, suggesting that the proposed method at 400 epochs or more had an overfitting problem. Therefore, 300 was set as the optimal number of epochs in this study.

The proposed method had high ACC1 performance at 0.90 or higher and improved the ACC1 on the subset of images acquired using devices from different vendors by incorporating changes in the magnification of width owing to changes in the patient’s table height in each examination as data augmentation. As shown in a previous study on the left–right flipping mistakes in chest X-ray images [45], data augmentation techniques that would result in undesirable transformations of clinical images, such as random flips (left/right or top/bottom), were not used in this study because there were concerns about an increased risk of human error.

For standard patients without implanted devices, as shown in Fig. 6a, regions of the mediastinum were extracted as patient characteristics. The training dataset contained images without implanted devices (the first image) and these (the second image), and the implanted devices in the validation image were not properly recognized as important areas for feature extraction (Fig. 6b). However, the patient shown in Fig. 6c had recognized implanted devices as important areas, and these were used as the patient characteristic features, because all training datasets contained implanting devices. Even if some patients were implanted with the same device, the implantation position and X-ray projection angle during scout imaging differed in each patient. Therefore, we believe that there is no issue of extracting the regions of the implanted devices in scout CT images as patient characteristics.

Our findings have important practical implications. First, a key feature of the proposed method is that re-identification can be performed with high accuracy, even for patient data that are not used in training. Similar to the re-identification models demonstrated for other medical images [2, 4, 12–24], our results demonstrate the power and potential of the proposed method for scout CT images of the trunk. Another study [2] used ACC10 as the threshold for alert occurrence with a value of 0.96. Using ACC10 as a threshold in this study, an alert could be triggered on 4% of top-10 error rate, even if the patient is correctly identified. A 4% erroneous alarm rate may seem high, given that the misfiling rate is 0.075% [3] or 0.002% [8]; however, we believe it is possible to re-assess whether the patient in the image is the correct patient with high accuracy by considering disturbing factors such as intra-patient variations in clinical application as shown in Table 4. When our framework is adopted for other scout CT image datasets in future research, the proposed feature extractor may be substituted with retraining (or transfer learning) for optimal usage. Second, our clinical dataset, split into training, validation, and test sets, reflected a real clinical scenario in a single center, where developers train models using retrospective images and test (clinical application) their performance on prospective images. Hence, we believe that the approach in this study will provide reliable assistance for preventing human error in clinical applications related to the re-identification of examined patients who underwent CT scans.

This study has certain limitations. First, the dataset was obtained using three CT devices: two scanners by SIEMENS and one scanner by GE. The number of images acquired with the GE scanner (1774) was fewer than that acquired with the SIEMENS scanners (7054), and there was a potential risk that learning was biased toward SIEMENS images. In addition, different CT devices, particularly those from other vendors, might have different imaging protocols and magnifications for scout CT images. An unknown domain, such as images acquired using the devices from other vendors, may affect patient re-identification performance, and the performance achieved in this study may not be reproducible. The re-identification performance of follow-up scans using devices from different vendors in this study was improved using the proposed augmentation, RandomTransversalScaling; however, the performance remained significantly lower than that of devices from the same vendor (*p* < 0.01). Therefore, in this study, a domain-shift problem is thought to exist for other CT vendors. Furthermore, previous studies have shown that it is possible to detect patient race, age, and view position using chest X-ray images [46–48]. Therefore, theoretically, scout CT images of the trunk might have domain-shift problems for identifying patient race, age, and view position or other domains. Second, the dataset of this study was based on a retrospective analysis, and patients younger than 20 years were excluded. Such patients may experience changes in their physical characteristics owing to growth between the two CT examination periods, which may affect the performance of patient re-identification. Third, this method only works for patients who underwent at least two scout scans; therefore, it cannot be applied to patients being examined for the first time.

Further research is required to evaluate how domain-shift problems, caused by intra-patient variations such as scan range, lung condition, intestinal gas, and arm position, as well as between scanners from different vendors, degrade biometric performance. Therefore, future research in this domain should use training datasets consisting of scout CT images obtained from scanners from all vendors or consider domain-adaptation techniques [25–28, 49–52].

## Conclusion

In this study, we proposed a patient re-identification method using a deep feature extractor that is robust even when applied to medical images obtained from devices from different vendors. The proposed method showed the possibility of linking correct metadata to a set of CT images of a trunk with lost or wrongly assigned metadata. Clinical CT examinations utilizing cutting-edge technologies (such as the technique presented in this study) provide a key solution to the inherent potential risks that can lead to medical malpractice.

## Data Availability

All scout CT images in this study are owned by Yamaguchi University Hospital, Yamaguchi, Japan, and cannot be made publicly available owing to patient privacy, its proprietary nature, and ethical concerns.

## Appendix

### Data Augmentation

For each image *I*(*x*,*y*) in the training dataset, an augmentation strategy was utilized to obtain the transformed image *I*(*x*′,*y*′).

### Random Perspective

A random perspective augmentation in this study performed the perspective transformation of the image with a probability of 0.1 and a distortion scale of 0.1 with bicubic resampling.

### Random Rotation

A random rotation augmentation in this study performed the rotation transformation with bicubic resampling by an angle selected from the range of − 5° to 5° randomly. Pixels outside the rotated region were assigned zeros.

### Random Transversal Scaling

Random transversal scaling is an image data augmentation method that randomly changes the scale of an image in the transverse direction within a specified range. The formulas used for transversal scaling are presented in Eqs. (1) and (2).

1 $$\left[\begin{array}{c}x^{\prime}\\ y^{\prime}\\ 1\end{array}\right]=\left[\begin{array}{ccc}{S}_{{\text{x}}}& 0& 0\\ 0& 1& 0\\ 0& 0& 1\end{array}\right]\times \left[\begin{array}{c}x\\ y\\ 1\end{array}\right]$$

2 $${x}^{\prime}={S}_{{\text{x}}}\times x,y^{\prime}=y$$

where *S*_x_ represents the scaling coefficient calculated using Eq. (3) for the original image width *W*, and the scaling term *S* is selected randomly from a specified range

3 $${S}_{{\text{x}}}=\frac{W+S}{W}$$

Pixels outside the post-scaling region were filled with zero.

### Center Crop

A center crop augmentation in this study performed cropping around the center of the image to a specified size, with columns and rows of 224 and 384, respectively.

## Code Availability

The code for the proposed method associated with the current submission is available at https://github.com/d83yk/dml-scout-ct-patient-re-id.
